# Knowledge, Attitudes, and Practices Regarding Contraceptive Methods Among Reproductive-Age Women: A Cross-Sectional Study

**DOI:** 10.7759/cureus.104099

**Published:** 2026-02-23

**Authors:** Prasenjithan Sanjayanthan Keerthi, M B Hayagrivas, Hardik Gupta, Emani S Reddy, Reeta Mahey, Ashish D Upadhyay, Neha Varun

**Affiliations:** 1 Medical School, All India Institute of Medical Sciences, New Delhi, New Delhi, IND; 2 Obstetrics and Gynaecology, All India Institute of Medical Sciences, New Delhi, New Delhi, IND; 3 Statistics, All India Institute of Medical Sciences, New Delhi, New Delhi, IND

**Keywords:** contraceptive awareness, contraceptive usage barriers, family planning, knowledge attitude and practice, public health, reproductive health

## Abstract

Introduction

According to the United Nations, the global population has escalated from 2.5 billion in the 1950s to eight billion in 2022, thus necessitating effective family planning. Despite various public health initiatives, misconceptions persist, thus requiring further exploration of contraceptive awareness.

Objective

This study primarily assessed the knowledge, attitudes, and practices toward contraceptive methods among reproductive-age women attending All India Institute of Medical Sciences (AIIMS), New Delhi, a tertiary care family planning clinic in North India. The secondary objectives included evaluating social factors influencing contraceptive usage, exploring reasons behind non-utilization, and identifying primary information sources among participants.

Material and methods

A cross-sectional survey of 400 women aged 17-49 years was conducted from June to November 2023 among attendees of a tertiary care hospital using hospital-based convenience sampling after obtaining informed consent. Data were collected using a 30-item questionnaire that underwent content validation through expert review by five specialists from the Department of Obstetrics and Gynecology at a tertiary care hospital in North India. Knowledge was assessed using a composite score (maximum score = 7), while attitudes and practices were evaluated using Likert-scale items. Data were analyzed using descriptive statistics, chi-square tests, and logistic regression. For logistic regression, the knowledge score was dichotomized using the median value as the cutoff to identify factors associated with contraceptive awareness.

Results

The mean knowledge score was 2.80 (SD = 1.59), indicating moderate awareness. Condoms and oral contraceptive pills were most recognized. Television (71.5%) and friends/relatives (68.5%) were primary information sources. Common misconceptions included fears of infertility (42%) and reduced sexual pleasure (30%). In multivariate analysis, women with two children had a significantly lower proportion of knowledge adequacy than nulliparous women (p < 0.001). Among users, 54% reported consistent contraceptive use unless planning pregnancy; main barriers were lack of information (50.5%) and fear of side effects (40.6%).

Conclusion

Although awareness of contraceptive methods among the study population was moderate, misconceptions and fears regarding their use persisted. These findings highlight gaps in accurate knowledge and underscore the need for targeted educational interventions, particularly among families with fewer children, with an emphasis on partner-inclusive counselling. As a hospital-based cross-sectional KAP study, the results provide a descriptive overview of prevailing knowledge, attitudes, and practices related to contraception and help identify common misconceptions that may hinder the use of contraceptives. These insights could help in aiding context-specific awareness programs aimed at improving contraceptive use and supporting informed reproductive choices.

## Introduction

The human population has grown exponentially from 2.5 billion (1950s) to eight billion (2022), posing a threat to available resources [1]. This surge has caused national governments to set up family planning goals to maintain population growth at a sustainable rate. Family planning plays a big role in achieving major Sustainable Development Goals (SDGs) set by the United Nations as a part of their 2030 Agenda for Sustainable Development [2]. Contraceptive measures and awareness will help us to achieve the majority of the SDGs within a stipulated period [3]. India was one of the forerunners in launching the National Program for Family Planning in 1952. The focus was shifted to reduce fertility rates through the National Population Policy 2000. These policies have helped bring down the average annual exponential growth from 2.16% in 1991 to 1.64% in 2011 [4]. It further decreased to 0.68% in 2022 [5]. While such enhancements have a clear-cut effect on reproductive health, there is still scope for improvement. Contraceptive prevalence and the unmet need for family planning prove to be the key indicators employed by the United Nations to assess reproductive health [6]. Many couples avoid contraception due to limited knowledge, inadequate means, and cultural or social taboos. The National Family Health Survey-5 (2019-21) reports a contraceptive prevalence rate of 67% among currently married women aged 15-49 years in India, while unmet need remains high among younger women, particularly among women aged 20-24 years (17.3%) [7].

Recognizing the growing need to track and advance contraceptive awareness, the present study was designed with the following objectives: The primary objective was to assess the knowledge, attitude, and practice about various available contraceptive methods among reproductive-age women attending a family planning clinic. The secondary objectives were to assess the impact of multiple social factors affecting the use of different available contraceptive methods, to study the reasons for not using them, and to study the sources from which maximum contraceptive knowledge is obtained among the participants.

## Materials and methods

Study design

Women who fulfilled the inclusion and exclusion criteria were invited to participate in the study. They were explained the study protocol, and were not given any incentives for participating in the study.

A cross-sectional questionnaire-based study was conducted for a period of six months (June 2023 to November 2023) among reproductive-age women who presented to All India Institute of Medical Sciences, New Delhi, a tertiary care referral hospital in North India. 

Ethical consideration 

The study was initiated after obtaining institutional ethical clearance (Institute Ethics Committee (IEC) number: 251/16.05.23). Written informed consent was obtained from all participants, and confidentiality of participant data was maintained.

Sample size calculation

After an extensive review of the literature, we found that the knowledge of contraceptive awareness in India varies from 46% in a cross-sectional study by Rao et al. (2,500 nursing mothers) [8] to 90.4% in a cross-sectional study done by Wani et al. among healthcare workers [9]. Taking the reference proportion of aware participants as 50%, with an error margin of 5%, the sample size calculated was 400.

\( n = \frac{4pq}{d^{2}} \)

\( n = \frac{4(0.5)(0.5)}{(0.05)^{2}} = 400 \)

Development and administration of the questionnaire

Through literature reviews and consultations with obstetricians and gynecologists, items for a contraceptive awareness questionnaire were compiled, and duplicate items were removed by consensus. The preliminary questionnaire underwent content validation through expert review by five experts from the Department of Obstetrics and Gynecology at a tertiary care hospital in North India, resulting in a final 30-item questionnaire comprising eight demographic items, nine knowledge items, 11 attitude items, and five practice items. Of the nine knowledge items, only seven were scored items, while two items did not have a correct or incorrect answer and were therefore not scored. The complete questionnaire is provided in the Appendix.

The questionnaire was developed in English and administered by trained OPD interviewers. For participants who could not understand the English questionnaire, interviewers verbally explained the questions in the local language based on their training, without using a formally translated version. All participants who provided informed consent completed the questionnaire. For attitude and practice items, participants who were unwilling to respond to specific questions were recorded as non-responses and were excluded from the analysis of those specific questions only, while their responses to other items were retained for the corresponding analyses.

Interviewer bias was minimized through a standardized recruitment and administration process, in which all eligible women attending the outpatient department were approached sequentially as they presented for OPD services. Participants who consented were administered the complete questionnaire. Data collection was carried out by trained interviewers following standardized procedures, under the supervision of an Obstetrics and Gynecology faculty member at the study site. To mitigate social desirability bias, interviews were conducted in a secluded setting adjacent to the OPD, ensuring privacy while maintaining accessibility. Responses were recorded anonymously using Google Forms, and no personal identifiers were collected.

Inclusion criteria

The inclusion criteria were women between the ages of 17 to 49 years (in their reproductive age group) presenting to the OPD in the Department of Obstetrics and Gynecology, who willingly gave their informed consent for the study.

Exclusion criteria

The exclusion criteria were women who were not willing to give their informed consent for the study or those who did not meet the above-mentioned inclusion criteria.

Statistical analysis

Data were entered in Microsoft Excel (Microsoft Corp., USA) and analyzed using descriptive statistics. Stata Statistical Software release 16 (StataCorp LLC, College Station, TX) was used for statistical analyses. Knowledge items were scored as 0 or 1 (with multiple-choice questions requiring all correct responses to be scored as 1), and a cumulative knowledge score was calculated (maximum score = 7). The knowledge score, being the dependent variable, was dichotomized into adequate and inadequate knowledge using the median value as the cutoff for regression analyses. Attitude and practice questions were assessed using a three-point Likert scale. Qualitative data were reported as absolute numbers and percentages, while quantitative data were presented as means ± standard deviation. Categorical variables were compared using the Kruskal-Wallis test, as appropriate. Univariate and multivariate logistic regression analyses were performed using forward stepwise inclusion of variables with p < 0.10 in univariate analysis, and p < 0.05 was considered statistically significant.

## Results

A total of 400 responses were collected. In instances where all 400 participants have responded to the question, the total number is reported as “N.” If fewer than 400 participants have responded, the number of respondents is reported as “n,” with the exact figure specified, and the corresponding percentages are calculated based on this “n”. Reduced response counts reflected either non-applicability of the specific question or non-willingness to respond to the question. In some of the questions, respondents were permitted to select more than one option.

Baseline demographics

The characteristics of the study population are summarized in Table 1. The majority of the respondents were in the age group 26-35 years (175 (43.75%)). More than half of the respondents had no children (205 (51.25%)), followed by 77 (19.25%) who had two children. A large proportion of the respondents were married (288 (72%)). The highest number of respondents were from the upper lower class (173 (43.25%)) (Modified Kuppuswamy Scale, 2022 [10]). About 144 (36%) of the participants resided in urban areas. The majority of the respondents followed Hinduism (336 (84%)).

**Table 1:** Baseline demographic characteristics of the participants

Demographic characteristic	N (%)
Age in years	
17-25	114 (28.50)
26-35	175 (43.75)
36-45	81 (20.25)
46-49	30 (7.50)
Marital status	
Married	288 (72.00)
Single/In a relationship	109 (27.25)
Widowed	2 (0.50)
Divorced	1 (0.25)
Number of children	
0	205 (51.25)
1	75 (18.75)
2	77 (19.25)
3 and more	43 (10.75)
Educational status	
Illiterate	14 (3.50)
Primary school	6 (1.50)
Middle and high school	78 (19.50)
Intermediate, graduate, and professional degree	302 (75.50)
Occupation	
Unemployed	264 (66.00)
Unskilled, semi-skilled, and skilled worker	30 (7.50)
Clerical, shop, farm, and semi-professional	27 (6.75)
Professional	79 (19.75)
Monthly family income	
Less than ₹27,882	154 (38.50)
₹27,883 - ₹69,534	118 (29.50)
₹69,535 - ₹1,85,984	84 (21.00)
More than ₹1,85,985	44 (11.00)
Socioeconomic class	
Lower	11 (2.75)
Upper lower	173 (43.25)
Lower middle	75 (18.75)
Upper middle	101 (25.25)
Upper	40 (10.00)
Place of residence	
Metropolitan	103 (25.75)
Urban	144 (36.00)
Semi-urban	93 (23.25)
Rural	60 (15.00)
Religion	
Hinduism	336 (84.00)
Islam	55 (13.75)
Christianity	2 (0.50)
Others	7 (1.75)

Knowledge regarding contraceptive practices

**Figure 1:**
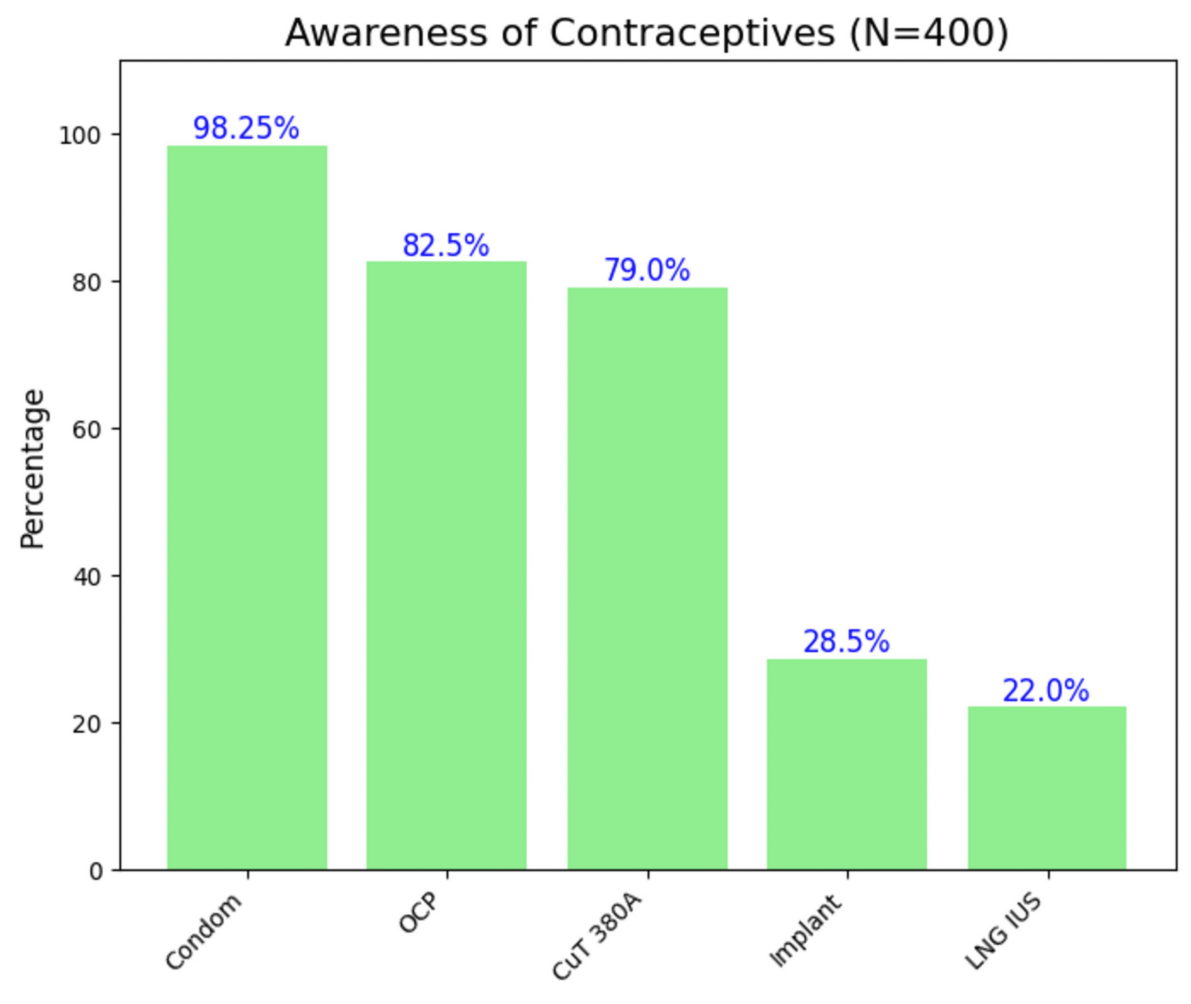
Awareness of contraceptives among reproductive-age women (N = 400, multiple-choice question) OCP: oral contraceptive pills; CuT 380A: copper T 380A; LNG IUS: Levonorgestrel Intrauterine System Image created by the author with Python version 3.10

**Figure 2:**
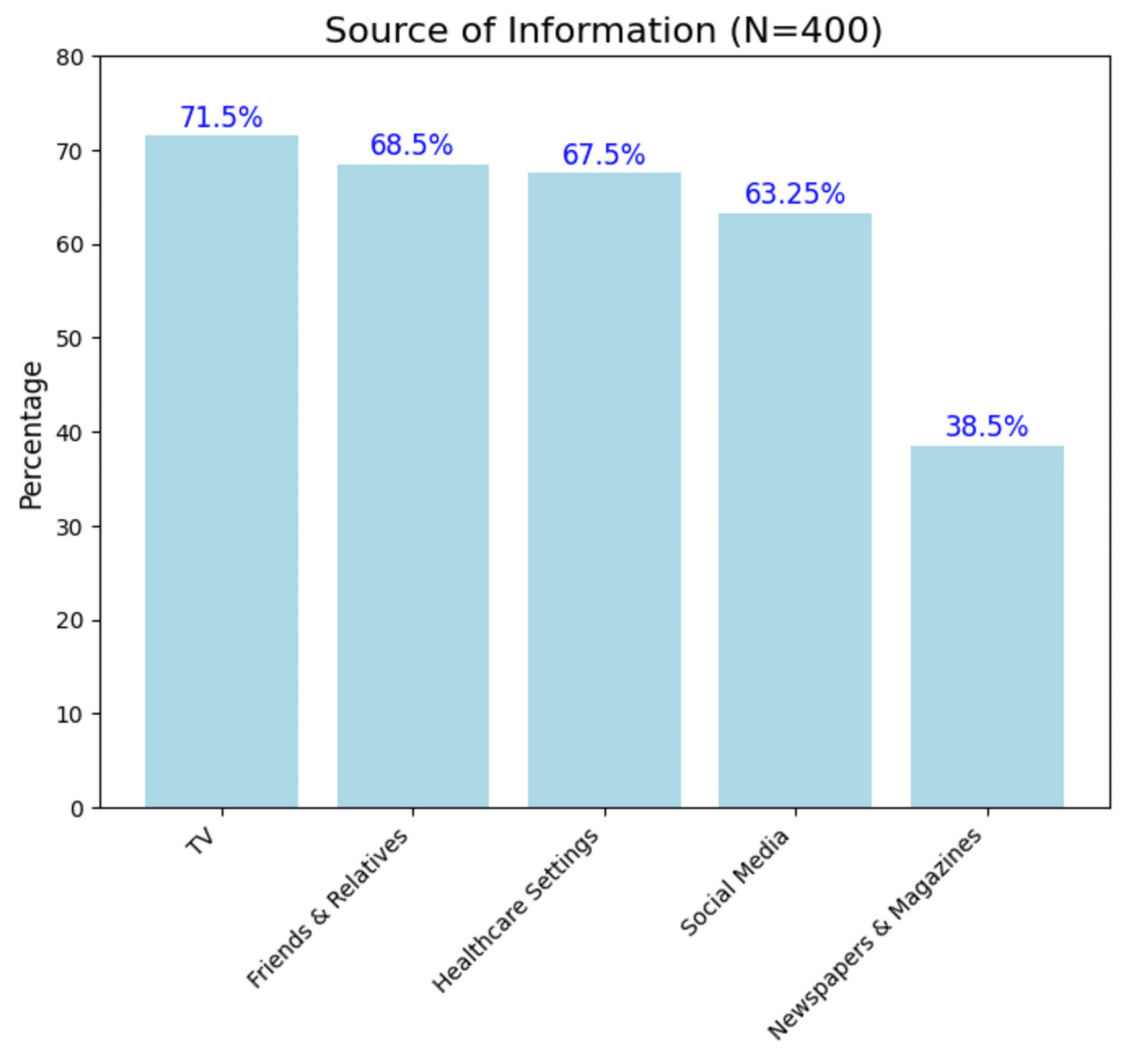
Source of information on contraceptives among reproductive-age women (N = 400, multiple-choice question) TV: television Image created by the author with Python version 3.10

The mean (SD) score was 2.80 (1.59) out of 7, with a median (IQR) of 3 (2-4). Most respondents (393 (98.25%)) knew about condoms (Figure 1). Television emerged as the source of information for awareness on contraception for the majority (286 (71.50%)) (Figure 2). While 247 (61.75%) participants believed that condoms were for men only, 122 (30.50%) correctly knew that both sexes could use them. Around half (201 (50.25%)) understood that condoms prevent pregnancy and STDs without hindering sexual pleasure. Only 57 (14.25%) knew that fertility returns within one to three months after stopping OCPs, and only 121 (30.25%) were aware of emergency contraceptives and their proper use, which were after a voluntary sexual act without contraceptive protection, after incorrect or inconsistent use of regular contraceptive methods, or in the case of contraceptive failure or mishaps. Only 108 (27%) knew that IUCDs are immediately effective, do not interact with other medications, and do not protect against STIs/HIV. Most knew that IUCDs, implants, and injectables as long-acting, reversible methods, while tubectomy is long-acting and irreversible (295 (73.75%)). In addition, 217 (54.25%) participants knew that tubectomy does not affect sexual pleasure.

The youngest (17-25 years) respondents (mean (SD) = 3.65 (1.50) and median (IQR) = 4 (2-5)) and the oldest (46-49 years) respondents (mean (SD) = 3.3 (1.44) and median (IQR) = 2.5 (2-5)) got higher scores (p = 0.0028). Women with no children had a mean (SD) score of 3.50 (1.48) with a median (IQR) score of 3 (2-5), and women with three or more children had a mean (SD) score of 3.14 (1.36) and a median (IQR) score of 2 (2-5) (p = 0.0412). Women who were single/in a relationship had a higher score compared to women who were married (mean (SD) score of 3.55 (1.51) and median (IQR) score of 3 (2-5) vs. mean (SD) score of 3.16 (1.38) and median (IQR) score of 2 (2 - 5)) (p = 0.0013). The results from the Kruskal-Wallis rank-sum test have been included to compare differences across groups in Table 2.

**Table 2:** Association between demographic variables and knowledge scores p-values were calculated using the Kruskal-Wallis test. The test statistic H follows a chi-squared distribution. A p-value < 0.05 was considered statistically significant.

Characteristic	N (%)	Mean score (±SD)	Median	Kruskal-Wallis test statistic - H	P-value (Kruskal-Wallis test)
Age (in years)				14.093	0.0028
17-25	114 (28.50)	3.65 (1.50)	4		
26-35	175 (43.75)	3.14 (1.40)	2		
36-45	81 (20.25)	2.91 (1.27)	2		
46-49	30 (7.50)	3.30 (1.44)	2.5		
Marital status				8.245	0.0412
Single/in a relationship	109 (27.25)	3.55 (1.51)	3		
Married	288 (72.00)	3.16 (1.38)	2		
Divorced	1 (0.25)	2 (0)	2		
Widowed	2 (0.50)	2 (0)	2		
Number of children				15.713	0.0013
0	205 (51.25)	3.50 (1.48)	3		
1	75 (18.75)	3.12 (1.34)	2		
2	77 (19.25)	2.79 (1.28)	2		
3 and more	43 (10.75)	3.14 (1.36)	2		
Education				1.185	0.7565
Illiterate	14 (3.50)	3.36 (1.50)	2.5		
Primary school	6 (1.50)	3.17 (1.47)	2.5		
Middle and high school	78 (19.50)	3.10 (1.41)	2		
Intermediate, graduate, professional degree	302 (75.50)	3.29 (1.43)	3		
Occupation				1.246	0.7419
Unemployed	264 (66.00)	3.30 (1.44)	3		
Unskilled, semi-skilled, and skilled worker	30 (7.50)	3.23 (1.33)	3		
Clerical, shop, farm, and semi-professional	27 (6.75)	3.18 (1.57)	2		
Professional	79 (19.75)	3.14 (1.38)	2		
Monthly family income				3.913	0.271
Less than ₹27,882	154 (38.50)	3.41 (1.45)	3		
₹27,883 - ₹69,534	118 (29.50)	3.19 (1.40)	2		
₹69,535 - ₹1,85,984	84 (21.00)	3.09 (1.35)	2		
More than ₹1,85,985	44 (11.00)	3.16 (1.54)	2		
Socioeconomic class				5.579	0.2329
Lower	11 (2.75)	3.54 (1.44)	3		
Upper lower	173 (43.25)	3.30 (1.44)	2		
Lower middle	75 (18.75)	3.24 (1.40)	3		
Upper middle	101 (25.25)	3.35 (1.47)	2		
Upper	40 (10.00)	2.78 (1.25)	2		
Place of residence				2.459	0.4828
Metropolitan	103 (25.75)	3.38 (1.46)	3		
Urban	144 (36.00)	3.15 (1.40)	2		
Semi-urban	93 (23.25)	3.16 (1.39)	2		
Rural	60 (15.00)	3.40 (1.49)	3		
Religion				3.377	0.3371
Hinduism	336 (84.00)	3.23 (1.42)	2		
Islam	55 (13.75)	3.45 (1.42)	3		
Christianity	2 (0.50)	2 (0.00)	2		
Others	7 (1.75)	3.28 (1.60)	2		

Predictors of the knowledge score

On univariable logistic regression, it was observed that as the age category increases, the proportion of knowledge adequacy in that category decreases. This can be seen in the example that people aged 17-25 years had a higher proportion of knowledge adequacy (baseline) than those aged 26-35 years (odds ratio = 0.54, p = 0.011) and those aged 36-45 years (odds ratio = 0.43, p = 0.004). The proportion of knowledge adequacy in married participants (odds ratio = 0.65, p = 0.058) was found to be less than those who were single/in a relationship (baseline). Participants who had no children were observed to have a higher proportion of knowledge adequacy (baseline) compared to those who had two children (odds ratio = 0.33, p < 0.001). On multivariable analysis, only the number of children predicted adequacy of knowledge, where people who had no children had a higher proportion of knowledge adequacy, compared to those who had 2 children (p = 0.0004) (Table 3).

**Table 3:** Univariable and multivariable analyses to assess the effect of baseline demographic parameters on knowledge scores Multivariable logistic regression models were developed by including all variables with p < 0.10 in the univariate analysis in a forward stepwise manner, and variables with p < 0.05 were considered statistically significant. For logistic regression analysis, the knowledge score was dichotomized using the median value as the cutoff. Boldface in the univariable panel represents p < 0.10, and in the multivariable panel represents p < 0.05. SD, standard deviation; 95% CI, 95% confidence interval. Univariate regression was not applicable for the 'divorced' and* 'widowed'* items due to the minimal number of responses in this group. Cells left empty under multivariate regression represent variables that were not eligible for carry-forward after univariate analysis.

Characteristic	Univariate odds ratio (95% CI)	Univariate p-value	Multivariate odds ratio (95% CI)	Multivariate p-value
Age (in years)				
17-25	Baseline odds			
26-35	0.54 (0.33-0.87)	0.011		
36-45	0.43 (0.24-0.76)	0.004		
46-49	0.65 (0.29-1.46)	0.3		
Marital status				
Single/In a relationship	Baseline odds			
Married	0.65 (0.42-1.01)	0.058		
Divorced				
Widowed				
Number of children				
0	Baseline odds			
1	0.72 (0.42-1.23)	0.23	0.62 (0.36-1.07)	0.088
2	0.33 (0.19-0.58)	<0.001	0.29 (0.16-0.52)	<0.001
3 and more	0.75 (0.39-1.44)	0.385	0.66 (0.33-1.32)	0.241
Education				
Illiterate	Baseline odds			
Primary school	1 (0.15-6.77)	1		
Middle and high school	0.73 (0.23-2.29)	0.594		
Intermediate, graduate, and professional degree	1.01 (0.35-2.96)	0.981		
Occupation				
Unemployed	Baseline odds			
Unskilled, semi-skilled, and skilled worker	1.13 (0.53-2.40)	0.759		
Clerical, shop, farm, and semi-professional	0.68 (0.30-1.51)	0.342		
Professional	0.78 (0.47-1.30)	0.344		

Attitude regarding contraceptive practices

**Figure 3:**
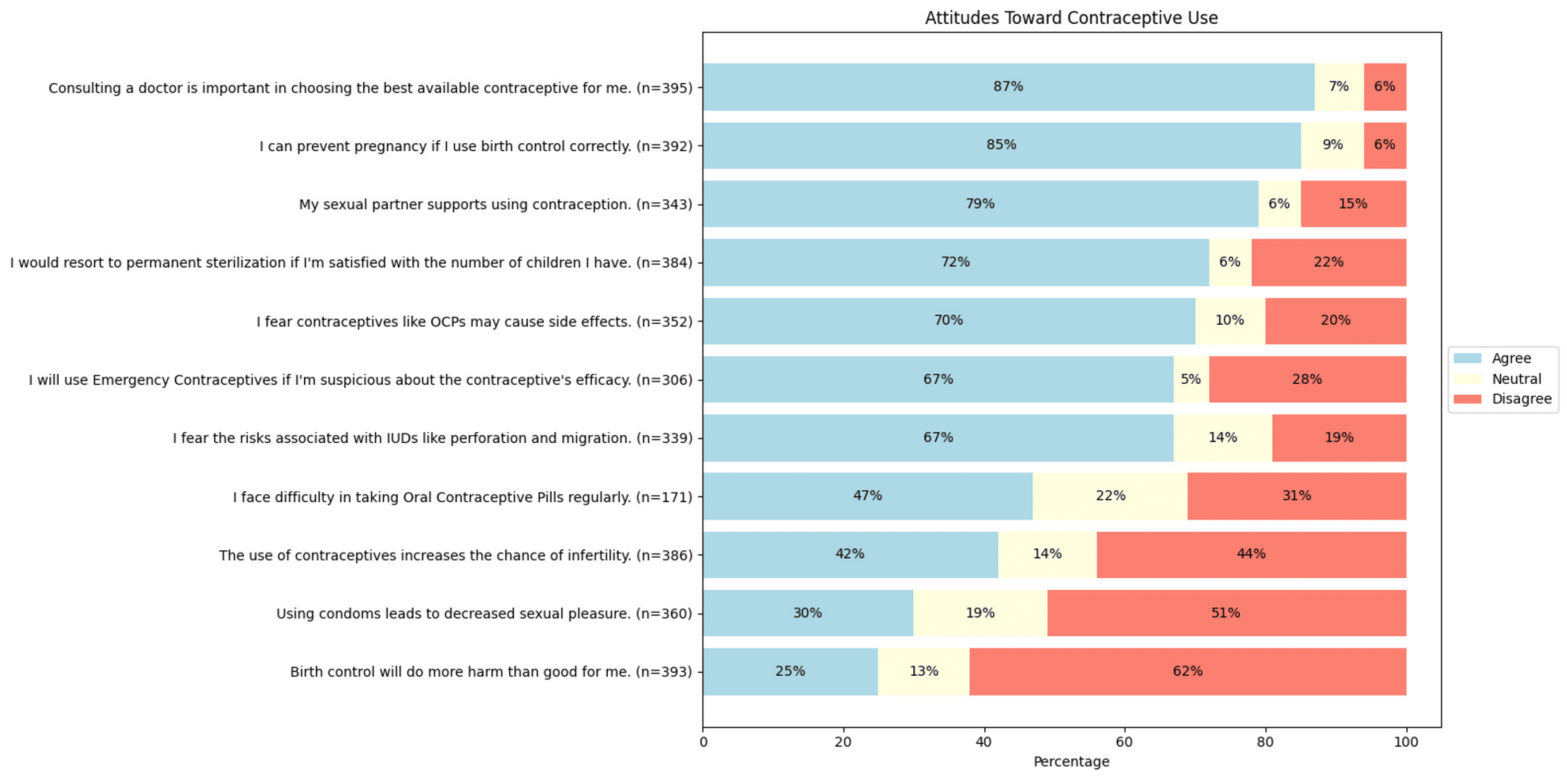
Attitudes toward contraceptive use in reproductive-age women OCP: oral contraceptive pills; IUD: intrauterine device Image created by the author with Python version 3.10

The descriptive analyses of the responses to the questions on the attitude domain are summarized in Figure 3. Most respondents (271 (79%, n = 343)) felt that their sexual partner would support contraceptive use. A majority (244 vs. 99 (62% vs. 25%, n = 393)) disagreed that birth control does more harm than good, and 334 (85%, n = 392) believed that correct use of birth control can prevent pregnancy. Around two-thirds (204 (67%, n = 306)) would use emergency contraception if they doubted a method’s effectiveness. Many feared the side effects of OCPs (245 (70%, n = 352)) and IUDs (228 (67%, n = 339)). While 162 (42%, n = 386) thought that contraceptives might increase future infertility, 169 (44%, n = 386) disagreed. Regarding condoms and sexual pleasure, 183 (51%, n = 360) disagreed that they reduce pleasure, while 109 (30%, n = 360) agreed. Most respondents, 276 (72%, n = 384), believed that they would opt for permanent sterilization once they had enough children, and 345 (87%, n = 395) viewed a doctor’s consultation as crucial in choosing the right contraceptive.

Correlation of the attitude of respondents with knowledge adequacy

The correlation of knowledge adequacy with the response category of individual attitude-based questions was assessed. On logistic regression, fear of risks associated with intrauterine devices, such as uterine perforation and migration into adjacent structures (e.g., bladder or peritoneal cavity), was significantly associated with higher odds of having adequate knowledge (odds ratio = 1.45, 95% CI (1.23-1.71), p < 0.001).

Practices followed regarding contraceptives

**Figure 4:**
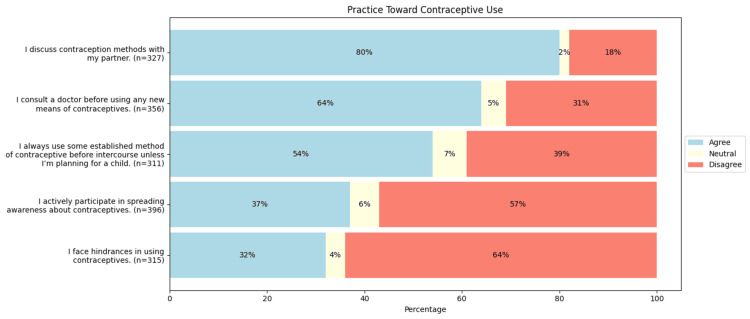
Practice toward contraceptive use in reproductive-age women Image created by the author with Python version 3.10

**Figure 5:**
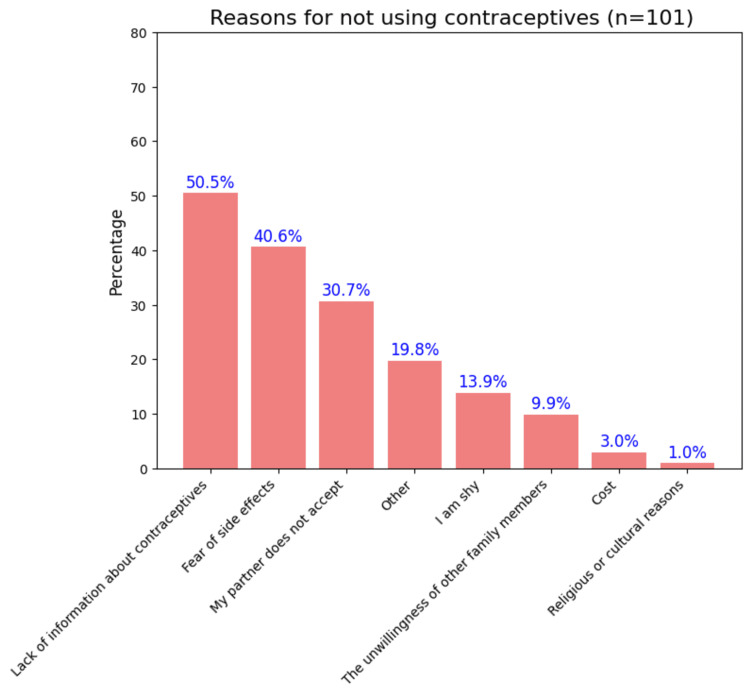
Reasons for not using contraceptives in reproductive-age women (n = 101, multiple-choice question) Image created by the author with Python version 3.10

The descriptive analyses of the responses to the questions on the practice domain are summarized in Figure 4. Of the total, 169 (54%, n = 311) consistently use contraception before intercourse unless planning a child, while 121 (39%, n = 311) do not. Most respondents (263 (80%, n = 327)) discuss contraceptive methods with their partners, and 228 (64%, n = 356) consult a doctor before trying new methods of contraception. More respondents (226 vs. 174 (56% vs. 37%, n = 396)) do not actively promote contraceptive awareness. Most (202 (64%, n = 315)) have no obstacles in using contraceptives, while 101 (32%, n = 315) face some hindrance. Among those encountering barriers, the most frequent reasons were lack of information (51 (50.50%, n = 101)) and fear of side effects (41 (40.59%, n = 101)) (Figure 5).

## Discussion

The findings of our study suggest a moderate level of contraceptive awareness among reproductive-age women in India. Awareness was highest for condoms, followed by OCPs and IUDs. Similarly, Choudhary et al. (2014, N = 400, pregnant females) in North India found that the highest awareness was of condoms (87.90%), then IUDs (71.00%) and OCPs (57.60%) [11]. Participants' knowledge of tubectomy was high in our study. However, there was a notable lack of awareness regarding long-acting reversible contraceptives (LARCs) such as IUDs, mirroring Muralidhar et al. (N = 303 pregnant tribal women) in South India, where 87.1% knew of sterilisation but only 39.3% knew of temporary methods [12]. Around 30.25% knew about emergency contraceptives, which is quite low and similar to Rahman et al. (N = 1474, reproductive-aged women) in Sikkim, where 40.6% had heard of them and only 19.6% had adequate knowledge [13]. Such disparities may reflect health system-level counselling practices that prioritize permanent methods after family completion, as well as persistent misconceptions regarding the safety, reversibility, and side effects of long-acting reversible options. These findings underscore the need for targeted educational efforts on all contraceptive options.

Television and friends/relatives were the primary sources of contraceptive information in our study, contrasting with Singh et al. in 2010 (N = 17,643, North Indian women), where husbands (43.5%) were the main source, followed by media (18.9%) [14]. Meanwhile, Pakrashi et al. (2022) showed that married women exposed to TV family planning campaigns have higher awareness, intention, and usage of modern contraception, stressing the need to continue and strengthen TV outreach [15]. However, reliance on informal sources can spread misinformation, as seen in our findings, where 162 (42%, n = 386) believed that contraceptives may cause infertility, and 109 (30%, n = 360) felt that they reduce sexual pleasure. Pal et al. (2021, N = 331, immediate postpartum women) similarly noted 7.8% avoided contraception fearing permanent infertility [16]. These recurring misconceptions suggest that information obtained through non-medical channels may be incomplete or distorted. Hence, credible channels like Accredited Social Healthcare Activists (ASHA) and the National Family Planning Program must address such myths. In our study, 67.5% got information from healthcare settings, lower than the >80% reported by Panda et al. (National Family Health Survey-5 data) [17]. Hence, healthcare providers need to play a more active role in educating the public about contraception by counselling them, irrespective of the complaints that bring them to healthcare providers.

After the Kruskal-Wallis and univariate regression analyses, younger and unmarried women had significantly higher knowledge scores (p < 0.1), contrasting with Muralidhar et al., who found that older women had greater odds of knowing at least one method of contraception (OR = 1.09, p < 0.2) [12]. Saya et al. in Puducherry (N = 2228) observed a significant association (p < 0.05) between maternal education and contraceptive awareness (adjusted prevalence ratio = 27.96), which our study did not replicate [18]. The number of children also influenced knowledge scores, where it was highest among nulliparous women, decreasing until parity two, then rising again. On multivariate analysis, only the number of children predicted the proportion of people with knowledge adequacy (p = 0.0004). Singh et al. noted a similar pattern. In their study, the regression coefficients for contraceptive knowledge/use were highest among women with no children (reference group), then decreased to 0.178 for women with two children, before increasing again to 0.328 among those with four or more children [14]. This U-shaped pattern might be because informed women often delay or limit childbearing, while those with three or more children may have already received hospital-based counselling. Hence, counselling on contraception should be more targeted to families having one or two children.

In our study, the majority of participants (79%) reported partner support for contraceptive use, which is higher than the 57.4% noted by Wani et al. [9]. Similarly, Sneha et al. (N = 209, reproductive-aged women) found that 79.45% of women were comfortable discussing contraception with their husbands [19]. Despite these encouraging trends, our study shows that among those encountering barriers, around a third of the participants cited partner non-acceptance. This indicates that while communication and support have improved over time, there remains a portion of couples where resistance persists. Consequently, it is crucial to involve both partners and, when possible, other family members in counselling and education efforts, ensuring misconceptions are addressed at the household level.

In our study, about half of the participants consistently used contraception unless they were actively trying for a child, a rate lower than reported by Rattan et al. (2022), Osborn et al. (2020), and Wani et al. (2019), which were 67%, 75% (95% CI: 73.6-76.4%), and 72.3%, respectively [9,20,21]. About 101 (32%, n = 315) of women faced barriers in using contraceptives, such as lack of information, fear of side effects, and partner non-acceptance. Yadav et al. (N = 535, women living in a slum) similarly noted that 55.1% did not use contraceptives due to insufficient knowledge [22]. Taken together, these findings indicate that inconsistent contraceptive use is shaped by a combination of informational, interpersonal, and contextual factors. Addressing these barriers may require more sustained, individualized counselling approaches that move beyond method awareness to actively engage with fears, misconceptions, and household-level influences that affect contraceptive behaviour.

Notably, the majority of our respondents feared OCP-related side effects and IUD risks. Interestingly, a higher proportion of knowledge adequacy predicted a greater fear of IUDs (p < 0.001). This prediction might be because those with greater awareness of contraceptive methods might also be more informed about their potential side effects. Other studies, such as Rattan et al. (2022), Mohapatra et al. (2021), and Yadav et al. (2016), also cite fear of side effects as a key reason for non-use [20,22,23]. Bhat et al in 2022 (N = 414, reproductive-aged women) found that 60.9% perceived OCPs as the most harmful contraceptive [24]. Hence, contraceptive awareness programs must be tailored to address the fear of side effects and to instill confidence in people who are not using contraceptives due to a lack of knowledge.

This study has certain limitations that should be acknowledged. As contraception is a sensitive and personal subject, participants may have modified their responses or have chosen not to answer certain questions due to social desirability bias, particularly in an interviewer-administered setting. Although the questionnaire was expert-validated, the absence of a prior pilot study may have limited the ability to assess the clarity and internal consistency of items before full-scale administration. In addition, the reliance on self-reported information introduces the possibility of recall bias. Furthermore, the cross-sectional design restricts the ability to infer causal relationships between socio-demographic factors and contraceptive knowledge, attitudes, and practices.

## Conclusions

Our study indicates that reproductive-age women in North India have moderate contraceptive knowledge, highlighting the need for targeted education on all available options. Our study identifies prevailing misconceptions, such as concerns about infertility and reduced pleasure, which may hinder the use of contraceptives. These misconceptions point to specific, modifiable gaps in health communication, particularly in how risks and side effects are discussed during routine counselling, and represent actionable targets for intervention. Despite the presence of numerous healthcare policies and programs, myths exist due to a lack of awareness. This suggests that the challenge lies less in policy availability and more in the effectiveness and consistency of information delivery at the point of care. To address these, healthcare providers and programs offering reliable information must actively involve the entire family, especially those with one or two children. Such family and partner-inclusive approaches may help translate awareness into sustained contraceptive use by addressing household-level decision-making. Awareness initiatives should focus on easing fears about side effects and building confidence among those who currently lack information. These findings can directly inform the design of targeted counselling strategies and mass communication efforts aimed at improving informed choice rather than method recognition alone. Moreover, our questionnaire can be applied in different settings and compared with our findings to identify both similarities and differences, guiding improvements in contraceptive education. Although the questionnaire was expert-validated, the absence of prior pilot testing should be considered when interpreting item-level responses; nevertheless, it offers a structured framework for future research and programmatic evaluations.

## Appendices

Study questionnaire

**Table 4:** Demographics questionnaire Credits: Prasenjithan Sanjayanthan Keerthi, M B Hayagrivas, Hardik Gupta, Reeta Mahey, and Neha Varun

Items	Response categories
Age in years	17-25
	26-35
	36-45
	46-49
Marital status	Married
	Single/in a relationship
	Widowed
	Divorced
Number of children	0
	1
	2
	3 and more
Educational status	Illiterate
	Primary school
	Middle and high school
	intermediate, graduate, and professional degree
Occupation	Unemployed
	Unskilled, semi-skilled, and skilled worker
	Clerical, shop, farm, and semi-professional
	Professional
Monthly family income	Less than ₹27,882
	₹27,883 - ₹69,534
	₹69,535 - ₹1,85,984
	More than ₹1,85,985
Place of residence	Metropolitan
	Urban
	Semi-urban
	Rural
Religion	Hinduism
	Islam
	Christianity
	Others

**Table 5:** Knowledge questionnaire *Multiple correct responses allowed Credits: Prasenjithan Sanjayanthan Keerthi, M B Hayagrivas, Hardik Gupta, Reeta Mahey, and Neha Varun

Items	Response options
1) Are you aware of any of these contraceptive methods?*	Condoms
	Oral contraceptive pills
	Implants
	CuT 380A (Copper T)
	LNG IUS (Levonorgestrel Intrauterine System, eg: Mirena)
2) During intercourse, who can use condoms?	Male
	Female
	None
	Both
	Not aware
3) What are the effects of condoms?*	Prevent pregnancy
	Protection from STDs (sexually transmitted diseases)
	Interferes with sexual pleasure
	Not aware
4) When does fertility resume after stopping the use of oral contraceptive pills?	Immediately
	1-3 weeks
	1-3 months
	Not aware
5) In what situations can emergency contraception be used?*	After a voluntary sexual act without contraceptive protection
	Incorrect or inconsistent use of regular contraceptive methods
	In case of contraceptive failure or mishaps
	Not aware
6) The following are the advantages offered by intrauterine contraceptive devices except:*	Is effective immediately after insertion.
	Does not interact with any medicines the user may be taking.
	Protects against STIs (sexually transmitted infections) / HIV (human immunodeficiency virus)
	Not aware
7) Which of these are your sources of information on contraceptive awareness?*	Social media
	Television
	Newspapers and magazines
	Friends and relatives
	Healthcare settings (hospitals and healthcare centres)
8) The following methods are long-acting reversible contraception except:*	Tubectomy
	Intrauterine contraceptive device (IUCD)
	Implants
	Injectables
	Not aware
9) What is the effect of tubectomy on sexual pleasure?	No changes in sexual pleasure
	Hinders sexual pleasure
	No response
	Not aware

**Table 6:** Attitude questionnaire IUD: intrauterine device, OCP: oral contraceptive pills Credits: Prasenjithan Sanjayanthan Keerthi, M B Hayagrivas, Hardik Gupta, Reeta Mahey, and Neha Varun

Items	Response categories
1) My sexual partner supports using contraception.	Agree
	Neutral
	Disagree
	No response
2) Birth control will do more harm than good for me.	Agree
	Neutral
	Disagree
	No response
3) I can prevent pregnancy if I use birth control correctly.	Agree
	Neutral
	Disagree
	No response
4) I will use Emergency Contraceptives if I’m suspicious about the efficiency of the contraceptive I used during sex.	Agree
	Neutral
	Disagree
	No response
5) I face difficulty in taking OCPs regularly.	Agree
	Neutral
	Disagree
	No response
6) I fear contraceptives like OCPs may cause side effects like weight gain, irregular menstruation, hypertension, etc.	Agree
	Neutral
	Disagree
	No response
7) I fear the risks associated with IUDs like perforation, migration (into the bladder, peritoneum, etc), and discomfort during insertion.	Agree
	Neutral
	Disagree
	No response
8) The use of contraceptives increases the chance of infertility in the future.	Agree
	Neutral
	Disagree
	No response
9) Using condoms leads to decreased sexual pleasure during sexual intercourse.	Agree
	Neutral
	Disagree
	No response
10) I would resort to permanent sterilization if I’m satisfied with the number of children I have.	Agree
	Neutral
	Disagree
	No response
11) Consulting a doctor is important to choose the best available contraceptive for me.	Agree
	Neutral
	Disagree
	No response

**Table 7:** Practice questionnaire *Multiple correct responses allowed Credits: Prasenjithan Sanjayanthan Keerthi, M B Hayagrivas, Hardik Gupta, Reeta Mahey, and Neha Varun

Items	Response categories
1) I always use some established method of contraception before intercourse unless I’m planning for a child.	Yes
	No
	Neutral
	No response
2) I discuss contraception methods with my partner	Yes
	No
	Neutral
	No response
3) I consult a doctor before using any new means of contraceptives.	Yes
	No
	Neutral
	No response
4) I actively participate in spreading awareness about contraceptives.	Yes
	No
	Neutral
	No response
5) I face hindrances in using contraceptives.	Yes
	No
	Neutral
	No response
5a) If yes, it’s because:*	I am shy
	My partner does not accept
	Religious or cultural reasons
	Cost
	Fear of side effects
	Lack of information about contraceptives
	The unwillingness of other family members
